# Utilization of a 3D Printed Orthodontic Distalizer for Tooth-Borne Hybrid Treatment in Class II Unilateral Malocclusions

**DOI:** 10.3390/ma15051740

**Published:** 2022-02-25

**Authors:** Andrej Thurzo, Wanda Urbanová, Bohuslav Novák, Iveta Waczulíková, Ivan Varga

**Affiliations:** 1 Department of Stomatology and Maxillofacial Surgery, Faculty of Medicine, Comenius University in Bratislava, 81250 Bratislava, Slovakia; bohuslav.novak@fmed.uniba.sk; 2 Department of Orthodontics and Cleft Anomalies, Dental Clinic 3rd Medical Faculty Charles University, Faculty Hospital Kralovske Vinohrady, 10034 Prague, Czech Republic; wanda.urbanova@gmail.com; 3 Department of Nuclear Physics and Biophysics, Faculty of Mathematics, Physics and Informatics, Comenius University, Mlynska Dolina F1, 84248 Bratislava, Slovakia; iveta.waczulikova@fmph.uniba.sk; 4 Institute of Histology and Embryology, Faculty of Medicine, Comenius University in Bratislava, 81372 Bratislava, Slovakia; ivan.varga@fmed.uniba.sk

**Keywords:** distalizer, dental photopolymers, orthodontics, biocompatible 3D printing, CAD/CAM, personalized treatment, device customization, computer modelling, unilateral class II

## Abstract

This paper introduces a novel method of 3D designing and 3D printing of a hybrid orthodontic tooth-borne personalized distalizer for treatment of unilateral Class II malocclusion. Research objectives were to clinically utilize 3D printed distalizers, appraise feasibility of this technique and compare two different biocompatible photopolymers (white and transparent). Frequency of distalizers’ debonding and patients’ aesthetical perception was evaluated on the set of 12 complete orthodontic treatments. The mean duration of treatment period with a bonded distalizer was 6.4 months. All cases were adults with unilateral Class II malocclusion managed with a hybrid approach as a part of Invisalign^®^ comprehensive treatment. Results showed that such perspective practice is feasible for 3D design and in-office 3D printing of a personalized distalizer. Results also showed no clinically significant differences between both studied biopolymers. The paper discusses an evaluation of such personalized distalizer functionality with regard to the current state of the art and compares to conventional prefabricated alternatives like a Carriere^®^ Distalizer™ appliance. Research showed a preference of patients towards transparent biocompatible photopolymer instead of the white A2 shade. The paper concludes that additive manufacturing from dental resins is a viable method in personalization and in-office 3D printing of orthodontic auxiliaries, particularly distalizers. New materials for orthodontic 3D printing endow enhanced individualization, thus more efficient treatment.

## 1. Introduction

Recent progress in digital workflows facilitated countless opportunities in customization of orthodontic devices. Advance of the material research of dental composites, including the dental composite resins intended for 3D printing, brought a rapid advance in more personalized and thus more effective orthodontic treatment. The last decade brought significant improvements of mechanical material properties as well as surface characteristics and biocompatibility of composites used in dentistry [1,2,3,4,5,6,7,8]. Focus on new bioactive compounds in composites to prevent dental caries development and progression has led researchers to the use of nanotechnologies [9,10]. Composite configuration has significant impact not only on 3D-printed appliance properties [11,12,13] but also on adhesion efficacy when used as adhesive for an appliance [14,15].

This paper introduces a novel approach based on 3D designing and printing of a personalized distalizer in Class II malocclusions, for which the prevalence and orthodontic treatment need are well studied [16].

Biomechanical principles of such 3D-printed personalized distalizer are not different from Carriere Motion 3D Appliances (CMA) even in combinations with CAT [17,18].

**Figure 1:**
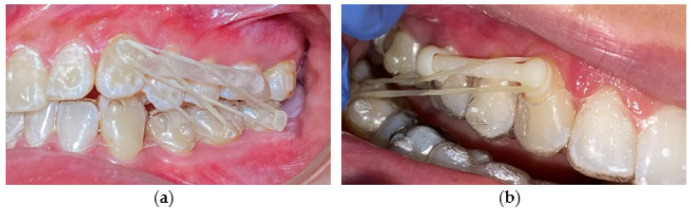
Examples of bonded 3D printed distalizers with an individual base for a tooth-borne hybrid approach in Class II unilateral malocclusions’ treatment and comparison of two biocompatible resins (**a**) transparent resin- Dental LT Clear V2; (**b**) opaque white Denture Teeth resin.

Various techniques have been introduced in orthodontic therapy of Class II malocclusions. In some protocols, maxillary molar distalization can be applied to correct molar relationships in orthodontic patients with maxillary dentoalveolar protrusion [19,20]. To prevent an unnecessary extraction of upper premolars, the upper molars can be distalized by means of orthodontic forces [21]. In addition, different techniques have been presented to reduce the dependence on patient compliance. However, all devices produce undesirable tipping of the maxillary molars as well as the loss of anterior anchorage to some extent during distalization movement [22,23]. The Carriere Motion Appliance (CMA) in the treatment of Class II malocclusion results mostly in dentoalveolar effects [19,23]. Patients today pursue orthodontic treatment with a desire for an aesthetic and comfortable alternative to conventional fixed appliances [24]. CAT was introduced as a reaction to this demand. The patient compliance is the prime weakness of CAT. A 3D printed “CMA-like” distalizer concept intended as a tooth-borne fixed appliance bonded with adhesive on tooth enamel surface is shown in Figure 1a,b.

It is known that maxillary molar distalization up to 2.5 mm is one of the most predictable movements with CAT [19,25,26]. Such significant predictability can be achieved with arrangement of an intelligent aligner sequence, the use of an appropriate attachment arrangement, and full-day Class II elastics [19,26]. CAT that includes difficult tooth movements like significant distalization is even more sensitive to patient compliance [27].

Class II treatments with CAT require mean treatment times of approximately 20 months during which Class II elastics must be utilized all day from treatment beginning until the class I canine relationship has been achieved [19,28]. Auxiliary devices (like distalizers) should be comfortable, provide rapid and effective treatment, and favour patient compliance with such hybrid orthodontic treatment. This is one of the reasons this paper presents the assessment of two different biocompatible materials—a clear (transparent) composite resin and an opaque white (A2 shade) resin. Clear aligners are aesthetically acceptable and comfortable [19,29,30], albeit the patient compliance is found to be better in the early stages of CAT [19,31]. With understanding of the importance of patient compliance during CAT, its weakness linked with removability and the advantage of fixed orthodontic devices for particularly difficult tooth movements, we can summarize:

1. patient compliance can be improved with aesthetical and comfortable devices;
2. combination of CAT and fixed orthodontic devices can result in optimized patient adherence to therapy, reducing the time required to wear Class II elastics;
3. treatment effectivity can be improved by the employment of a fixed appliance.

This combined clinical approach has been named hybridization of aligner therapy [19]. Among others, temporary tooth-borne distalization devices are the most popular hybridization approaches in CAT. Typically, they are not intended for the whole duration of the orthodontic treatment.

**Figure 2:**
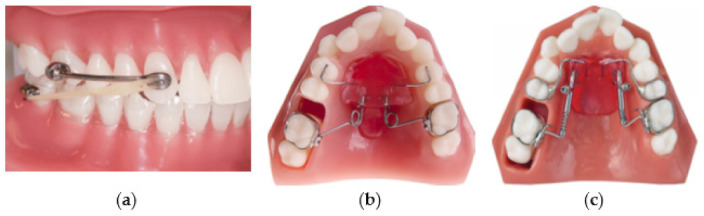
Examples of molar distalization appliances—distalizers (**a**) Carriere Motion 3D Appliance (CMA); (**b**) Pendulum appliance; (**c**) Distal Jet appliance.

Several types of molar distalization appliances (distalizers) are presented in the literature, such as the Carriere Motion 3D Appliance (CMA) (Henry Schein Orthodontics, Carlsbad, CA, USA), the Pendulum device, and the Distal Jet appliance (Figure 2).

Distalizers shown in Figure 2a–c are believed to be easy to mount intraorally and can promote distal movement of the maxillary molars. Nevertheless, most of these distalizers show undesirable reciprocal loss of anchorage in the premolars and incisors during distal molar movement [19,32,33]. Furthermore, molar tipping is frequently observed in most of the cases. The Distal Jet appliance (Figure 2c) is composed of two bilateral tubes joined to a Nance appliance [19]. On the tube, there is a stainless-steel coil spring and a clamp that can slide toward the molar and be tightened to compress the coil. The force exerted by the spring begins at 150 g and decreases as space is opened [34]. The Pendulum appliance (Figure 2b) introduced by Hilgers in 1992 [35] is still one of the most used distalizing devices. It is a fixed appliance composed of a plastic pad in contact with palatal rugae. Both the Distal Jet and Pendulum produce an increase in the face vertical dimension due to a backward rotation of the mandible [19,36,37,38]. These vertical changes comprise a slight opening of the mandibular plane angle and an increase in lower anterior facial height [19,39]. Ghosh and Nanda reported that the increase in lower anterior facial height was significantly greater in patients with higher pre-treatment mandibular plane angles [19,40]. CMA (Figure 2a) consists of a rigid bar attached to the maxillary canine and first molar. Posteriorly, the pad with a ball-and-socket joint is bonded to the molar at the centre of its clinical crown to facilitate molar derotation and distalization. The frontal canine pad with a built-in mesial hook used for placement of intermaxillary elastics is bonded to the anterior third of the clinical crown [19,41,42,43]. The activation of CMA is usually obtained using two types of elastics: the first one being 0.25 in, 6 oz; the second one 0.19 in, 8 oz, to be used from the second month of treatment until the molar and canine class I relationships are established [44]. The principle of this appliance is like a cantilever-based fixed appliance previously shown by Nanda [45] who described the system as an effective way to correct molar Class II in nongrowing patients [19].

Previous retrospective clinical studies demonstrated the possibility of obtaining a maxillary molar distalization between 1.6 and 5.1 mm [44] with treatment time mean duration of about 4 to 5 months [19,44]. When compared to other Class II correction methods, CMA showed the same results obtained with Class II elastics in terms of molar distalization but in less time [46]. One clinically important effect of treatment with CMA appeared in the lower anterior facial height—an increase in the mandibular plane angle [19,47]. The results of the study published in The Angle Orthodontist showed that the CMA is an effective way of correcting the sagittal component of Class II malocclusion within the first half-year of treatment. Proclination of the lower incisors resulting from the Class II elastics mechanics was also observed and resulted in a significant amount (4 degrees) [19,41].

All the tooth-borne appliances mentioned above create some side effects that need to be controlled during the hybrid aligner therapy. Research published by Khosravi et al. [48] regarding overbite management with Invisalign aligners indicated that overbite correction is mostly related to anterior teeth movement without any significant posterior intrusion or extrusion [19,48]. In addition, Ravera et al. described the bite block effect of the aligners causing an intrusive effect on posterior teeth of 0.5 mm [19,22]. These findings were confirmed by Mantovani et al. [49].

The Carriere^®^ Motion 3D™ appliance (CMA; Henry Schein Orthodontics, Carlsbad, CA, USA) has become more popular during the past decade as a versatile intermaxillary Class II corrector. CMA was introduced by its developer, Luis Carriere, in 2004 as the Carriere^®^ Distalizer™, and the renamed Carriere^®^ Motion 3D™ appliance consists of two rigid bars bonded bilaterally to the maxillary canines and first molars. Regarding the long-term treatment stability: dental and skeletal treatment results of distalization accomplished with the Carriere Distalizer appliance followed by fixed appliance therapy displayed minor changes four years after treatment [50,51,52]. Publication about CMA modifications from 2021 from Wilson et al. in The Angle Orthodontist came to the conclusion that shortly CMA achieved Class II correction similarly to the standard CMA, with less change in overjet and distal tipping movement of the maxillary canines. The straightforward design makes CMAs more comfortable than the Forsus appliance [53,54]. The unilateral application of the Carriere Distalizer is possible and can be utilized for non-extraction Class II treatment [41,51]. We know that the CMA corrects Class II malocclusion through distal tipping and rotational movement of maxillary canines and molars and corrected mesial tipping of mandibular molars [55]. Material research of dental resins for 3D printing in orthodontics was mostly oriented on 3D printing of clear aligners [56,57,58]. Only recently, a wider range of Class IIa biocompatible dental resins for 3D printing has been introduced and fully certified in Europe. The goal of the procedure introduced in this paper is the materialization of personalized shape of biomechanical orthodontic auxiliary (distalizer) to increase the speed or comfort of the orthodontic treatment. Such 3D printed distalizer is unique in every patient, with appropriate length, curvature and most notably its terminal pads with individualized bases for bonding.

The main purpose of this paper was to show the entire procedure of 3D designing, printing and application of distalizer (CDA-like) for hybrid Class II CAT. The secondary objective was to compare two popular biocompatible materials and evaluate their clinical performance as well as the patient appraisal. The major conclusion of this paper is that an individual distalizer can be designed and materialized with little effort, achieving clinical objectives and high patient comfort.

## 2. Materials and Methods

### 2.1. Digital Workflow for Personalized 3D Design and Printing

**Figure 3:**
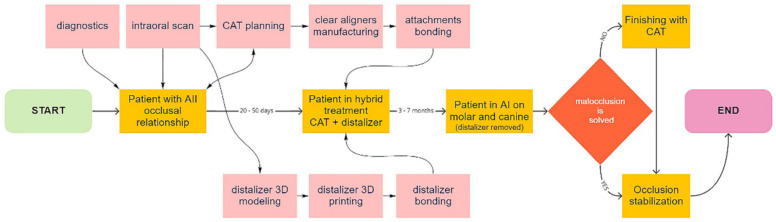
Scheme describing new digital workflow for orthodontists utilizing certified biocompatible dental resins for 3D printed personalized distalizers for hybrid treatment.

The biocompatible Additive Manufacturing (AM) brings better personalization of various orthodontic auxiliaries like power-arms, power-caps, retainers, and many other applications. The scheme in Figure 3 describes the entire workflow.

Two photopolymers were evaluated in this research within the application of the described novel method of design and manufacturing for a personalized distalizer. Materials and distalizers were clinically evaluated on the complete distalization treatment procedures on 12 patients with a unilateral Class II occlusal relationship. Twelve patients were chosen upon strict criteria from the total set of 387 monitored patients. Material distribution was random. The objective for the strict criteria was the elimination of possible bias caused by clinical differences.

**Figure 4:**
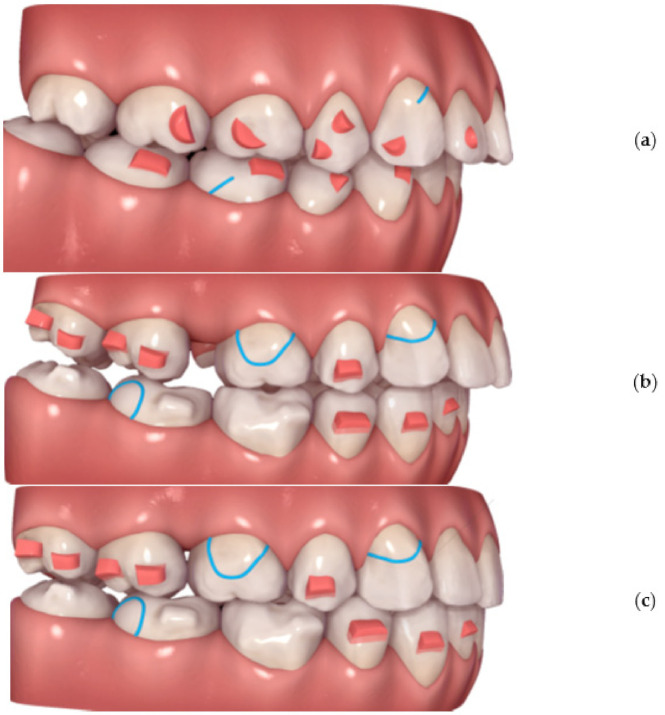
Planning of a personalized unilateral distalizer for upper teeth distalization in a clinical hybrid approach (**a**) initial setup with Class II. elastics in the CAT (Invisalign^®^) to achieve distalization of two terminal molars 18 and 17; (**b**) situation evaluated for a 3D printed personalized distalizer application with a similar biomechanical effect to CMA. The “Button cut-outs” in the aligner on teeth 13 and 16 are intended for 3D printed distalizer custom base fixed adhesion on tooth surface; (**c**) planned clinical target of hybrid treatment with CAT + distalizer with 15% overcorrection.

To professionally design, print and apply personalized distalizer for hybrid treatment, the more complex clinical picture and target shall be comprehended for each particular case (Figure 4). Example shows that not necessarily whole extent of distalization trajectory must be achieved only with a distalizer. Its combination with CAT allows for a wider scope of approaches for the orthodontist (Figure 1a–c).

### 2.2. Patients—Selection Criteria

**Figure 5:**
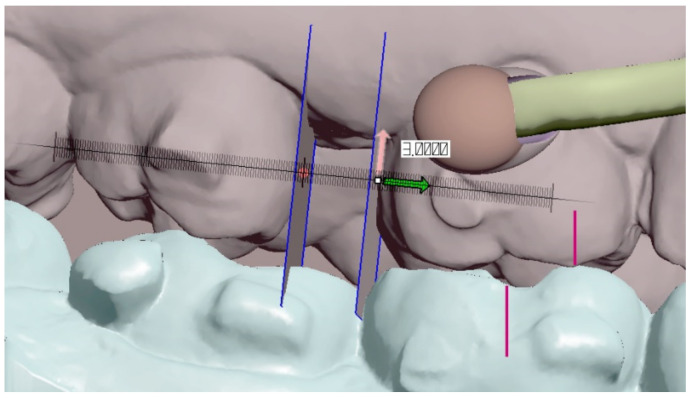
Digital measurement of planned clinical target (amount) of distalization in Autodesk Meshmixer—free software for working with 3D objects—in this case, the digital intra-oral scan.

To correctly compare both materials clinically, the strict criteria were defined to minimalize possible bias. Bias could result from a non-homogenous group affected by growth or skeletal discrepancies or extreme extents for distalization or application of different types of CAT or poor monitoring caused by patients’ non-compliance. Patients for evaluation of the described tooth-borne hybrid treatment approach with Class II unilateral malocclusions were chosen by the following selection criteria:

1. Patients must be adults;
2. Unilateral Class II occlusion measurable on molars and canines with a class I relationship on the opposite side and no skeletal Class II values (Figure 4);
3. Skeletal cephalometric values of SNA = 81 ± 3°, SNB = 78 ± 3°, ANB = 3 ± 2°;
4. Target of planned linear distance of the frontal cusp of upper molar translation (distalization and distorotation) had to be between 2.5 and 6 mm (Figure 5);
5. Patients were compliant with dental monitoring on a weekly basis;
6. Patients had a distalizer combined with CAT (all patients were using an Invisalign type of appliance).

The exclusion criterium for skeletal Class II was based on published variability of distalizers’ effects in between skeletal Class I and Class II cases. In addition, exclusion criterium for planned extent of upper molar distalization over 6 mm and under 4 mm allowed better harmonization of patient samples. Differences in distalization difficulty or the distalization setup would be the source of significant bias between both compared groups.

After application of these criteria from 385 A.I. monitored patients, only the final 12 adults in skeletal Class I that were acknowledged with Invisalign treatment were selected as suitable. These patients were randomly divided into two groups according to the type of dental resin used for distalizer manufacturing. All patients were monitored on a weekly basis by video-scans evaluated by an artificial intelligence algorithm and no significant differences in compliance were observed in these patients. Dental monitoring was able to identify immediately a drop or damage of orthodontic auxiliary (distalizer) so we were not dependent on patient self-reporting. The follow-up duration varied in patients as each case required a different amount of distalization under specific clinical anatomy.

### 2.3. Materials, Handling, 3D Printing and Post-Printing Protocols

Two long-term biocompatible composite resins, intended for 3D printing, were chosen to be compared:

1. Dental LT Clear Resin (RS-F2-DLCL-02 from Formlabs) [59,60,61];
2. Denture Teeth Resin (FLDTA101,PKG-RS-F2-DT Formlabs/Dentca) [61,62,63,64].

The reason for choosing these two materials was to evaluate if such a small difference of material properties does have significant impact on the clinical performance of 3D printed distalizer. The second reason was to evaluate the patient feedback for whether the transparent or the opaque distalizer is considered more aesthetical.

Dental LT Clear Resin (RS-F2-DLCL-02 from Formlabs) [59] is designed for hard splints and retainers with high resistance to fracture and wear. This resin is Class IIa biocompatible. 3D printing layer accuracy of this material is only 100 microns, which is sufficient for expected details of the distalizer; however, it was relevant to compare to second material, which was printed on a resolution of 50 microns. This biocompatible photopolymer resin is intended for a Form 2–3D printer. The advantage of this material is that it is highly durable and resistant to fracture and has high optical transparency, with resistance to discoloration over time. 

#### Post-Processing after 3D Printing

After printing the distalizer, it must be washed, and the support removed. Dental LT Clear Resin V2 shall be washed in IPA with a concentration of 99% or higher to comply with biocompatibility regulations. In our protocol, we have washed the clear distalizer parts for 15 min, then removed and soaked in fresh IPA for 5 min. For optimum mechanical properties, it must not be washed for more than 20 min. After washing, it must be light cured ideally in the Form Cure that helps 3D printed parts achieve their highest possible strength and stability. Optimal post-curing settings depend on equipment and the geometry of the part. In our case, the recommended settings can be applied [61]. This means that the distalizer from material Dental LT Clear V2 shall be light cured for 60 min in temperature of 60 °C. This cure setting ensures that it achieves both biocompatibility and optimum mechanical properties. This material has been updated in 2020 and clinicians shall remember this. The FLDLCL02 resin (available since 6/2020) is a new version of FLDLCL01 (was available since 10/2017). This new resin was used in this research and, in comparison to an older version of this composite material, it has reduced yellowing, improved durability and higher resistance to fracture. Further technical details about this material are summed up in Table 1 in addition to its technical and safety sheets online [59,62]

Denture teeth Resin (FLDTA101, PKG-RS-F2-DT from Formlabs/Dentca) [60,61,63].

Formlabs Denture Resins are certified biocompatible materials for 3D printing digital dentures and their parts. This white resin is also Class IIa biocompatible. 3D printing layer accuracy of this material is 50 microns, which allows double accuracy compared to previous transparent resin and better for expected details of the distalizer. Upon research and sending an inquiry to the official Formlabs support, we have learned that this resin is exceptionally not their proprietary and its original manufacturer is Dentca, Inc. located in Torrance, CA, USA. Composition of this resin matches DENTCA Denture Teeth resin, an FDA cleared biocompatible photocurable material with performance and strength matching conventional acrylic. DENTCA is a manufacturer of medical and healthcare devices focused on denture base and teeth products and was the first to receive FDA approval for such 3D printing resins.

Form wash setting for this application were different than in previous material. In previous transparent resin, the wash time was 20 min; in this denture-teeth resin, it is only 10 min. 

Form Cure settings for this material is specific. In our protocol, we have followed the official guidelines [63,64], and we have used a glass container with glycerine. After preheating the glycerine to 80 °C in Form Cure, we have used our own heat resistant to keep the distalizers fully submerged in the glycerine and kept the container inside Form Cure for 30 min. After the first 30-min post-cure, we have flipped the small distalizers to the opposite side and Post-cure again for 30 min. 

Composites are complex materials and finding the right one for our specific application requires balancing multiple attributes. Distalizer is a small part with a need for rapid prototyping. Mechanical properties of Denture teeth resin are Flexural Strength >65 Mpa (method ISO 20795-1) and density of the material of 1.15 g/cm^3^ (method ASTM D792-00). The Denture Base ISO Standard used is EN-ISO 20795-1:2013 (Dentistry—Base Polymers—Part 1: Denture Base Polymers), which is summarized in Table 2.

Material properties can vary with part geometry, print orientation, print settings and temperature. Data in Table 1, Table 2 and Table 3 refer to post-cured properties obtained after exposing parts to 108 watts each of Blue UV-A (315–400 nm), in a heated environment at 80 °C (140 °F) and 1 h, with six 18 W/78 lamps (Dulux blue UV-A) [59,60,61,62,63,64,65].

### 2.4. 3D Printing and Costs

3D stereolithography printer—Form 2 (Formlabs) was used for 3D printing with a standard setting. Its technical parameters are available online [65].

In every case, we have been printing on the highest resolution available (0.1 mm for clear or 0.05 mm for white resin). In every case, we have printed three clones in total, for practical reasons anticipating the situation of possible damage to the distalizer, to be able to immediately replace the damaged/debonded part. For simplicity, the shade of Denture teeth resin was chosen as A2. 

The approximate costs regarding prefabricated “CARRIERE Motion 3D Class II Appliances Distalizer” on eBay is $50–$60. Printing costs per one triple-set of distalizers is approximately $15 ($3–$5 a part). This calculation is approximation and relates to denture teeth resin costs, which are $399 per litre. Dental LT clear also costs $399 per litre. Costs for 3D printing with both materials do not differ significantly and neither do material mechanical properties.

A toothbrush wearing test was performed by Dentca company to review the abrasion resistance of preformed DENTCA Denture Teeth compared with commercial artificial polymer teeth. Relative worn volume % of ΔV to reference artificial acrylic tooth (100%) was 70%. This means that the DENTCA Denture Teeth are less worn than the commercial artificial acrylic tooth. Stain Resistance was assessed by Dentca to review the staining resistance of preformed Denture Teeth resin compared with commercial artificial polymer teeth by soaking into two different solutions. Staining resistance of DENTCA Denture Teeth is equivalent to an artificial polymer tooth.

Despite the resin manufacturing company, Dentca does not publish detailed material properties of their 3D printing denture teeth resin; it has been fortunately well researched [5,66]. Yoo-Jin Chung et al. in their article “3D Printing of Resin Material for Denture Artificial Teeth: Chipping and Indirect Tensile Fracture Resistance” evaluated chipping and indirect tensile fracture resistance of this resin.

Both materials have similar properties; however, they differ significantly in their clinical appearance (one is opaque white another is transparent). Participating patients were questioned about their satisfaction with distalizer appearance/aesthetics. Patients at the end of the treatment had an option to reference if the next distalizer would prefer to be the same colour or if they would prefer another (still choosing only between white and transparent).

### 2.5. Method of Digital Design of the Personalized Orthodontic Distalizer

**Figure 6:**
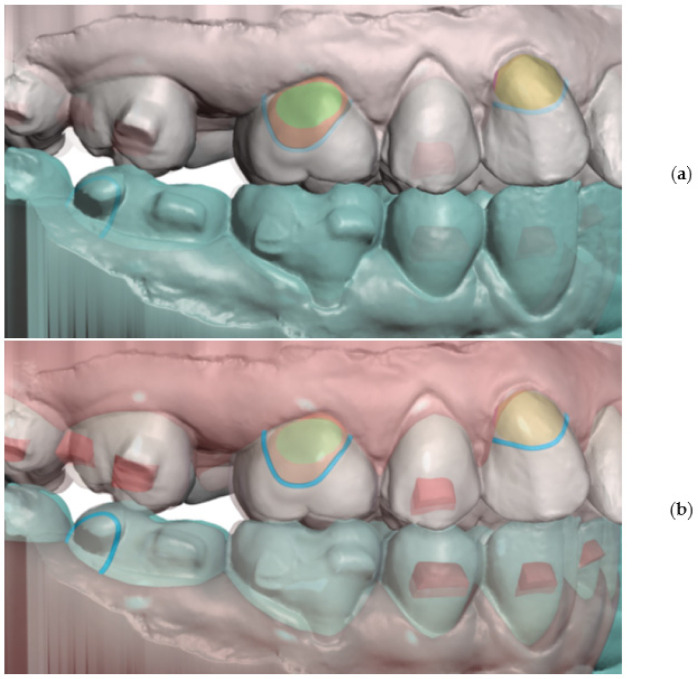
Overlay of intraoral scan (**a**) with a software model from CAT planning (**b**) is important to identify the areas of aligner cut-outs. These will be the contacts of distalizer with teeth surfaces.

One of the goals of this paper is to demonstrate that the making of personalized distalizer is not complicated and time-consuming. The whole hybrid treatment workflow is shown in the scheme in Figure 3. For designing a personalized orthodontic distalizer, we have followed these steps:

- The first step is to register an intraoral situation with a digital intraoral scan and exporting it in a common 3D format. For example, the STL file format is suitable and can be processed in various simple 3D modelling programs like Meshmixer (from Autodesk).
- The second step is to coordinate the distalizer and CAT planned effects and plan the placement of distalizer in harmony with CAT. In this research, all CAT were performed using Invisalign appliances (Figure 6).
- The third step was to design the body of the distalizer, including movable joints and individual bases on the terminal pads of the distalizer.
- The fourth step was clinical application and distalizer activation followed by weeks of movement Dental monitoring.
- The fifth step was performed after achieving planned occlusal correction. Removal of the distalizer was followed by stabilization of the result.

Planning of the complex hybrid treatment requires coordinating the distalizer and CAT. Their effects shall be in synergy and the placement of distalizer might be on teeth covered with aligner or, less frequently, not. One of the possible steps is overlay of intraoral scan with the CAT treatment model to identify the planned aligner cut-outs for 3D printed distalizer adhesive individualized bases (Figure 6). In this paper, we explain the advantage of a customized distalizer for a particular clinical situation (unilateral Class II) and particular clinical approach (tooth-borne hybrid treatment with an orthodontic distalizer and CAT).

**Figure 7:**
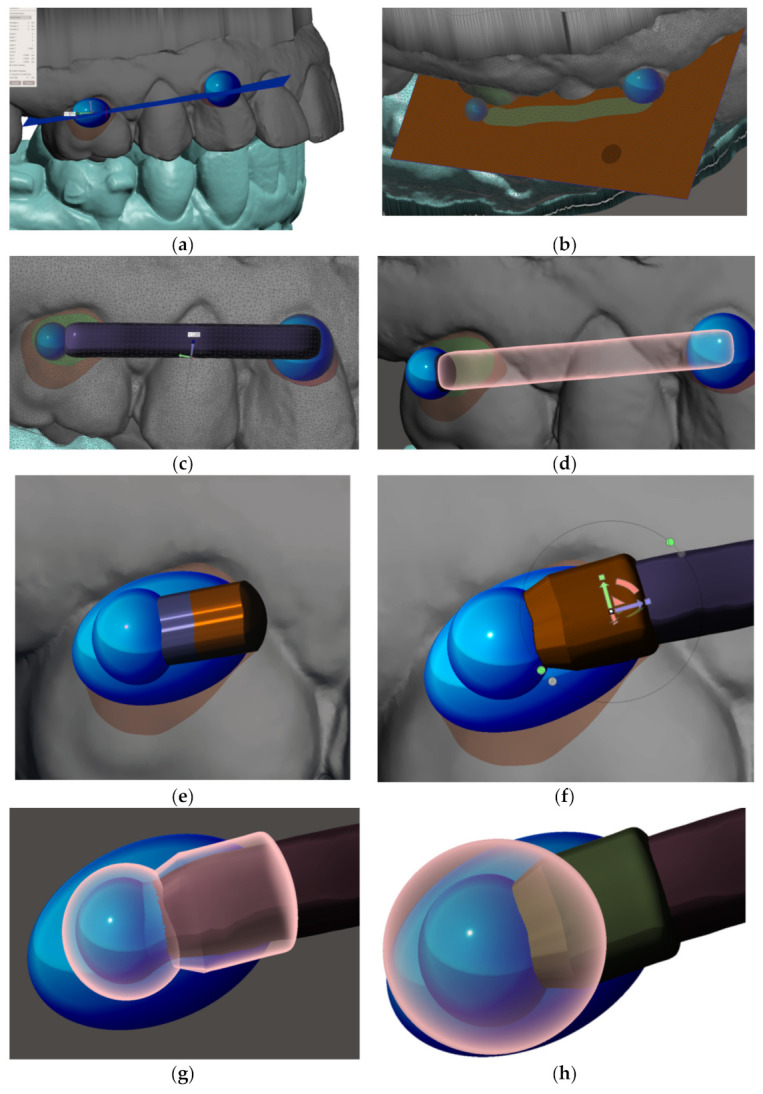
Method of designing personalized distalizer. (**a**) placing a plane for the upper border of arm of the distalizer and spheres overlaying teeth surfaces in the areas of future individualized adhesion pads; (**b**) optimized border and deleting the rest of the plane; (**c**) 4.5 mm extrusion of the 2D plane selection creates the arm of distalizer; (**d**) new sphere (d = 5 mm) on the distal end of the arm represents the terminal joint of the distalizer’s arm. New sphere, with the same centre is created with 0.1 mm bigger diameter and represents the future socket for the joint; (**e**) extrusion of the spherical socket for the joint in the direction of the arm, liberating its movement during distorotation of the molar; (**f**) extending the hiatus of the distalizer’s arm access to the joint socket; (**g**) visualization of the future joint socket and its access together with a distalizer’s arm distal ending with a spherical joint; (**h**) creation of final coverage of the joint with a new sphere resized as necessary to cover the joint socket at least by 0.6 mm.

After identification of the areas for distalizer bonding, the body, joint and bases shall be designed. To start designing the distalizer, the plane shall be defined on which the distalizer long axis will lie crossing the middle of the areas intended for bonding of the distalizer. Placing a 2D plane in the program like Meshmixer is sufficient. Placing and reshaping two spheres in these spots will represent future bodies of the individualized pads with bases copying the surface of the teeth in these locations (Figure 7a). After placements and resizing the spheres, a contour of the distalizer body shall be drawn on the plane, keeping a distance from premolar teeth surfaces of around 0.4–0.8 mm (Figure 1b).

After definition of the arm’s 2D contour (Figure 7b) extrusion follows. Subsequently with smoothing and rounding the edges of a created bar (in the Meshmixer program by pressing Ctrl + F), an arm of the distalizer is formed (Figure 7c). It can be directly merged with the future adhesive base on the canine. Another step is creating a new sphere with a diameter of 5 mm with a centre identical to the centre of the terminal sphere for joint of the distalizer’s arm. This sphere will represent the terminal joint socket for the distalizer’s arm. The difference between both spheres (joint head and joint socket) shall be between 0.1 mm up to 0.2 mm. New sphere, created will be extruded in arm accessing the joint (Figure 7e,f). Spherical socket is extended in this direction to allow the distalizer’s joint movements, liberating the arm during distorotation of the molar (Figure 7g). The final step in designing the juncture of both parts of the distalizer is the creation of final coverage of the joint with a new sphere resized as necessary to cover the joint socket by at least 0.6 mm (Figure 7h).

**Figure 8:**
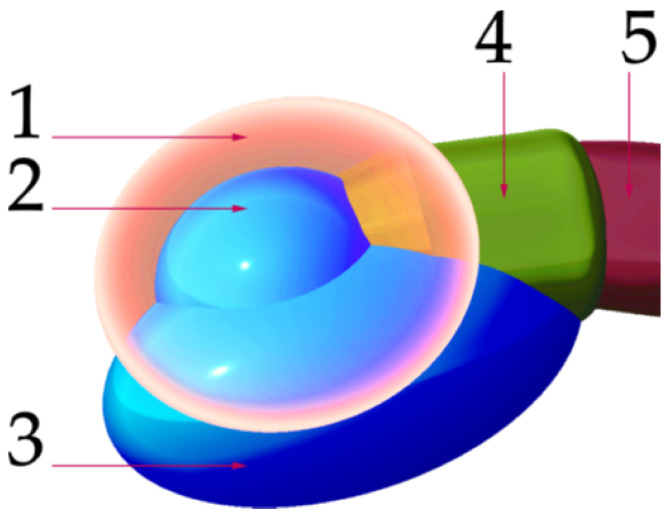
Main parts of the junction of both distalizers parts located on the smaller part intended for molar adhesion. (1) final encapsulation of the joint when merged with the base; (3) the body of the smaller part of the distalizer with a surface complementary to the surface of the molar, thus creating an individual base; (2) the socket of the joint; (4) extended neck of the accessory channel for the arm ending in the socket, liberating arm orientation during distalization and distorotation of the molar; (5) terminal part of the distalizer’s arm entering the socket with a sufficient gap around it;.

The scheme in Figure 8 describes the main parts of the juncture of both distalizers parts located on the smaller part of the distalizer intended for molar placement. (1) is the final encapsulation of the joint when merged with the base (3). This creates the body of the smaller part of the distalizer, intended for bonding on the vestibular surface of the molar crown. Part (2) represents the socket of the joint, where the pressure of the distalization is applied, allowing the distorotational movements of the molar. The last part (5) represents a curved terminal part of the distalizer’s arm entering the socket with a sufficient gap around it.

**Figure 9:**
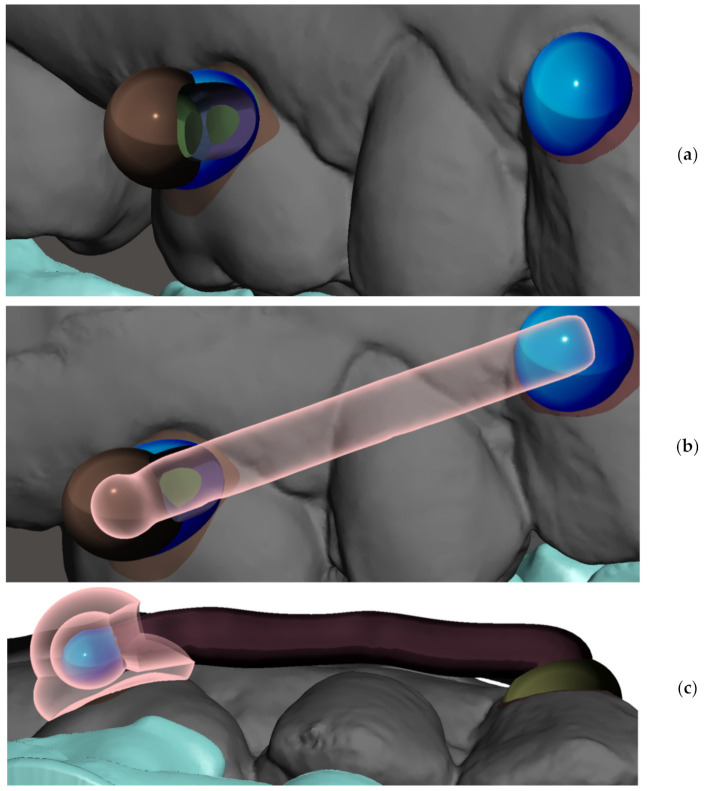
Visualization of the juncture of both parts of the distalizer (bigger part with the arm and joint and the smaller part with the socket). (**a**) visual of the molar part of the distalizer including the socket; (**b**) visualization of ending of the arm of the distalizer in the body of the distal part; (**c**) profile of the layers of the distalizer’s body, socket and the joint.

Figure 9 visualizes the final merge of the distalizer parts, creating individualized bases, arm, and the socket in regard to the teeth (Figure 9a–c).

**Figure 10:**
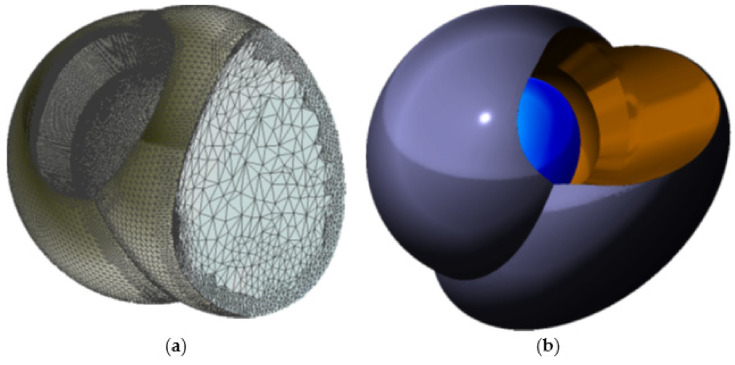
Vestibular and lingual side of the molar part of distalizer. (**a**) Wireframe appearance of the individual base that matches the tooth surface in the exact area that is helping in the exact positioning. (**b**) The tunnel that leads to the socket with a visible hiatus—a small circular step that is causing a “click-in” function, where slight pressure is necessary to “click-in” the arm of the distalizer with a spherical joint into the socket—both parts of distalizer shall be connected before bonding.

The individual base of the distalizer is responsible for a perfect match to the tooth surface. Both ends of the connected distalizer are intended to be bonded as a fixed orthodontic auxiliary for a necessary period of time during orthodontic treatment. Figure 10a shows how such an individual base looks. Figure 10b shows a hiatus in the tunnel leading to the socket. A small circular step results in a “click-in” function, where slight pressure is necessary to “click-in” the arm of the distalizer with a spherical joint into the socket.

**Figure 11:**
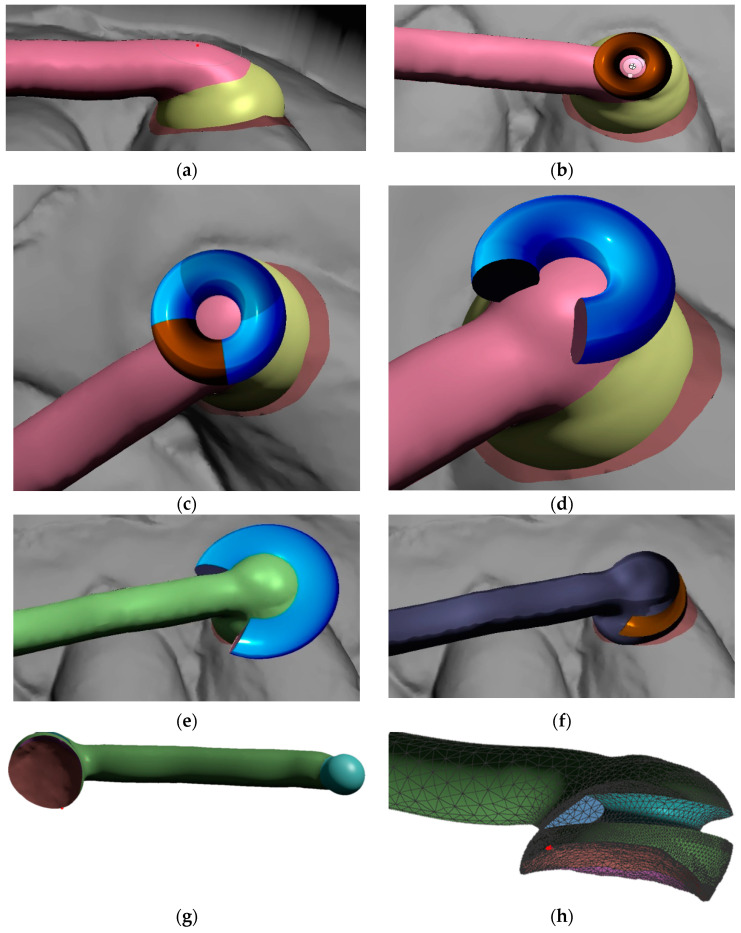
Creation of feature supporting anchorage of the elastic bands. (**a**) Step in connecting area between distalizer’s arm and the canine base is smoothed—different from Figure 9c. The purpose is better comfort and resiliency during chewing; (**b**) Import of a primitive donut shape from basic Meshmixer gallery allows faster subtraction of desired shape for loaded elastic band; (**c**) Quadrant not necessary for elastic retention shall be preserved to increase the strength of this part of the distalizer; (**d**) Reshaping of the reduced donut intended for Boolean subtraction (Select both objects and apply Boolean Difference in a precise Solution mode, preferably using Intersection curves, otherwise default setting in Meshmixer) Quadrant not necessary for elastic retention shall be preserved to increase the strength of this part of the distalizer; (**e**) before subtraction position properly so the long axis of future loaded elastic is pointing towards the lower anchor point—button or precision cut; (**f**) result of Boolean difference subtraction of the adapted donut shape; (**g**) result of Boolean difference between intraoral scan and the canine part of the distalizer—base of the canine pad is now fully individualized; (**h**) profile view of the mesial end of distalizer with the groove intended for the elastic band.

Further steps explained in the Figure 11a–h describe a creation of feature supporting anchorage of the elastic bands. Figure 11a shows a more rounded surface in contrast with Figure 9c. Such slight adaptations can be made wherever an operator considers it appropriate with the goal to achieve a more comfortable shape for the patient and better resiliency during chewing. The authors of this paper used the Meshmixer program from Autodesk and mode Sculpt → Surface → brush (robust-smooth). The step in connecting area between distalizer’s arm and the canine base is smoothed.

#### Elastic Bands Attachment and Distalizer Activation

Placement and removal of the elastic bands are performed by the patient. The elastics shall be changed for new ones at least three times per day. Activation of the distalizer is done immediately after bonding. The activation is obtained using two types of elastics: the first month 0.25 in, 6 oz; and 0.19 in, 8 oz, to be used from the second month of treatment. 

Loading with these heavy-force elastic bands (6 oz and 8 oz) is tooth borne from the pad on the maxillary canine to a mandibular molar button or precision cut in the lower aligner. 

A full-time wear from the patient (22 h) is expected. There are various ways of creating a button or another form of retentive feature on the canine pad. Figure 11 shows only one of them. This method and design of attachment for an elastic band are presented in Figure 11b–f. We consider very practical, comfortable and resilient. Figure 11g shows the arm of the distalizer with the individual base intended for the canine mesial–cervical part of maxillary canine.

**Figure 12:**
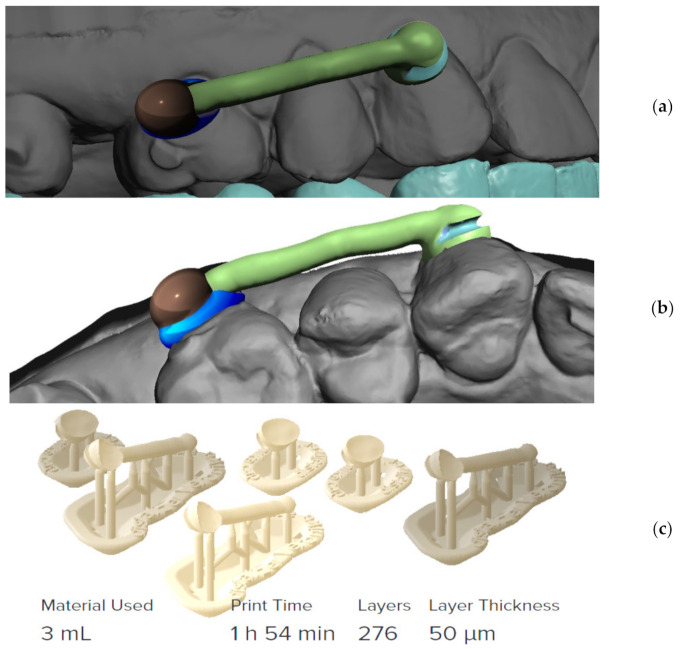
Final distalizer before 3D printing; (**a**) lateral view; (**b**) apical view; (**c**) 3D printing set-up for 50 µm printing resolution with visible supports; three clones were always printed for possible servicing reasons, material Denture teeth, shade A2.

Final distalizer is ready for 3D printing as a separate 3D layer in the program and is exported to 3D printing program as a separate STL file (Figure 12).

### 2.6. Practical Aspects of Post-Prints Handling

**Figure 13:**
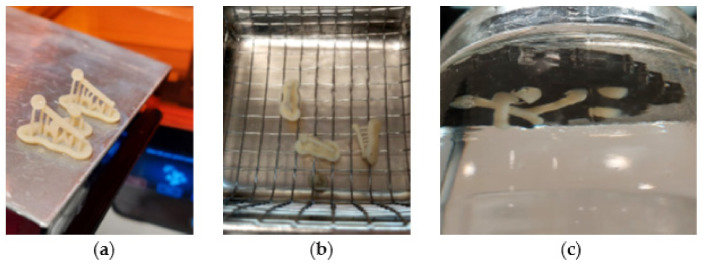
(**a**) Fresh 3D printed three clones of a distalizer from Denture teeth resin material; (**b**) sonic IPA bath for 10 min; (**c**) post-curing submerged in glycerine in 80 °C.

**Figure 14:**
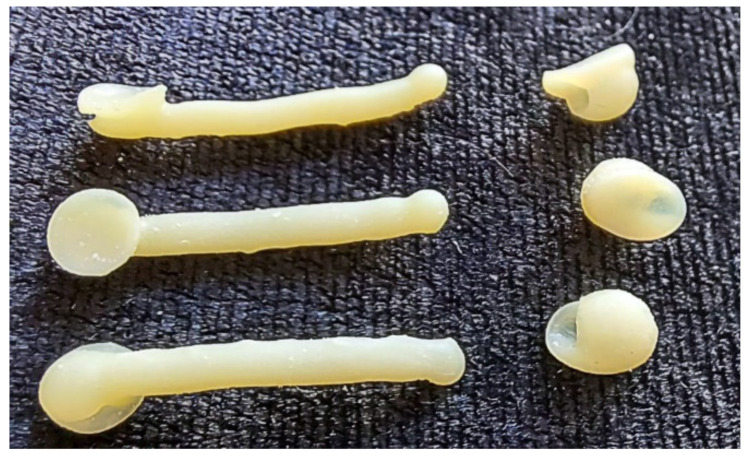
Three clones of a 3D printed distalizer from material Denture Teeth, shade A2.

**Figure 15:**
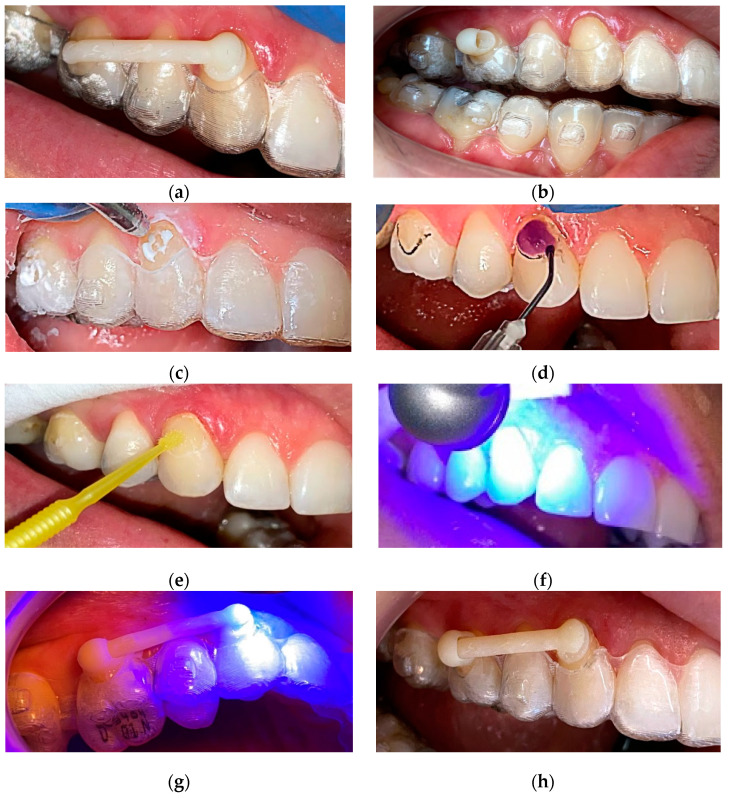
Clinical application of a personalized 3D printed distalizer in combination with CAT (Invisalign^®^); (**a**) Initial “try-out” of the mesial part of distalizer with its “arm”; (**b**) “try-out” of the distal smaller part of distalizer; (**c**) circular sandblasting 5 s each of the surface; this is proven to significantly increase bond adhesion strength [67]; (**d**) standard enamel etching with 37.5% phosphoric acid, rinsing and drying; (**e**) application of 7th generation Single Bond Universal from ESPE; (**f**) Standard Light curing of the bond; (**g**) Standard Light curing for final adhesion of the distalizer with a micro layer of 3M™ Transbond™ PLUS Color Change Adhesive; (**h**) final appearance of the distalizer before activation.

**Figure 16:**
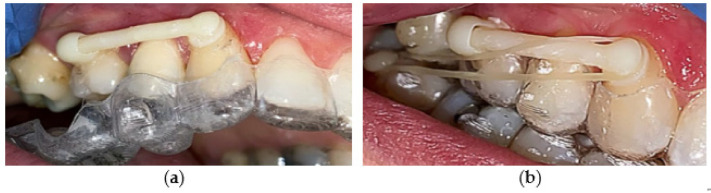
3D printed distalizer. (**a**) fixed distalizer in hybrid treatment with CAT (Invisalign^®^); before activation; (**b**) fixed distalizer in hybrid treatment with CAT (Invisalign^®^); after activation.

Post-prints handling with visual explanations are shown in Figure 13, Figure 14, Figure 15 and Figure 16. Figure 13 shows a 3D printed example of the distalizer designed in the previous chapter. The material Denture teeth Resin Formlabs/Dentca [60,61,63] requires washing in IPA for only 10 min (Figure 13a,b). We have used a sonic IPA bath for 10 min. The second step is post-curing of the prints; for this material, it is quite specific. In our protocol, we have followed the official guidelines [63,64], and we have used a glass container with glycerine. After preheating the glycerine to 80 °C in Form Cure, we have used our own heat resistant gear to keep the distalizers fully submerged in the glycerine during post-curing. We kept the container inside Form Cure for 30 min as prescribed. After the first 30-min post-cure, we have flipped the small distalizers to the opposite side and post-cured them again for another 30 min (Figure 13c). It is important to remember that the glycerine during this process will get very hot (80 °C), and heat resistant silicone tongs shall be used to insert and remove the distalizers from the solution.

Figure 14 shows a sharp contrast zoomed view of post-cured distalizers from Denture Teeth resin material in shade A2. The surface shows remnants of the supports, and these can be polished by a doctor during clinical application if necessary. Two of the clones are spares.

### 2.7. Method of Clinical Application

Clinical application takes advantage from the individualized base on each terminal pad of the distalizer. The surface of such individualized base exactly matches the tooth surface in pre-planned particular position and orientation. Figure 15 describes the sequence of trying out, sandblasting, etching and final bonding of the distalizer.

The last step before distalizer activation is to remove possible excessive remnants of the adhesive with the dental bur softer than enamel and harder than the polymerized Transbond™ glue. Removal of upper aligner is followed by the cleaning procedure (Figure 16a). The planned gap for the adhesive between tooth surfaces and the distalizer is usually 50 microns.

The last step is activation of the distalizer, which means that the forces from the elastics will be applied. The activation is obtained by the use of two types of elastics: the first month 0.25 inch (6.35 mm), 6 oz (170 g); and, from the second month 0.19 inch (4.8 mm), 8 oz (227 g), to be used from the second month of treatment. Figure 16b shows the distalizer with an elastic band from the canine pad to the button on tooth 47.

## 3. Results

The main goal of this paper was to introduce and practically accomplish the novel approach of a 3D printed distalizer in hybrid orthodontic treatment. This has been achieved and is documented in the procedure description in the previous chapter.

The secondary objective was to evaluate if any of the tested photopolymers is inappropriate for this intention, showing significantly more frequent debonding or breakage. Table 4 shows this evaluation of the set of 12 complete treatments with the described method, where two groups of six patients were treated with the distalizer from one of the observed biocompatible photopolymers. To complete a statistically more useful, larger set of treatments under such strict election criteria, it would require many more years. Only standard testing of the significance of the difference is feasible. The interpretation of the test results would be statistically better on a larger dataset; however, the time necessary for such extensive treatment in combination with clinical selection criteria (unilateral distalization) would require many more years of observation. As the pace of development of advanced photopolymers in dental 3D printing domain is quick, years-long research would most probably bring information on already obsolete and discontinued dental materials.

The results of the classic testing showed no significant findings due to the small numbers. Results are shown in the summary in Table 5. There is a combination of descriptive statistics and testing (only those *p*-values without an asterisk are “outcomes”). Poisson regression is a regression in which the dependent variable has a Poisson distribution (number of events in each region or a given time interval).

The results on debonding characteristics and their distribution in patients show seven failures in total. Characteristics are:

- A total of seven failures consisted of four debonding incidents and three breaks of material;
- Only in two patients did double failure happen (one debonding and one breaking);
- Both patients with double failures preferred the same material in the final question;
- Another three failures were separate incidents in separate patients.

**Figure 17:**
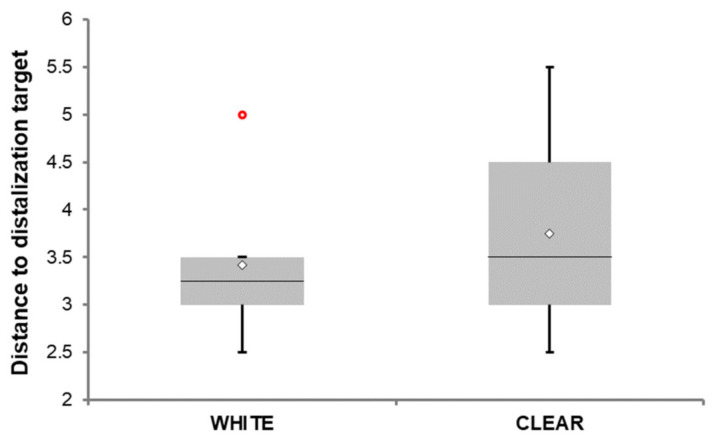
Data distribution of distance to distalization target stratified by type of photopolymer. Data are presented as the median (horizontal line) and mean (diamond sign), the lower-upper quartile range, minimal and maximal values. The red circle denotes an observation with a large but legitimate value that fell outside of the presumed normal distribution. A two-sample t-test verified by its non-parametric alternative was used to test the between-group differences in the distance. The difference between group means was not statistically significant (*p* = 0.578). Finally, tertiary objective was to examine patients’ preference after they had completed treatment with either white or clear (transparent) material. Only two patients out of a total of 12 patients (16.7%) preferred white resin over the transparent one. The question was “Which of these two materials would you consider more aesthetically appeasing for a distalizer if you can choose?”. They were shown examples of both materials together.

Figure 17 shows the extent of distalization for both groups. The range of data shown in Figure 17 may be viewed as an estimate of the range of possible outcomes, and the difference between two mean data points on the graph as an estimate of the expected difference between these two materials (the estimates were made using bivariate analysis, i.e., without taking into account the influence of other factors on the outcomes). Medians are depicted as lines within the boxes and the respective mean with a diamond sign. The “whiskers” extend from the ends of the box to the minimum and maximum data values. The circle denotes an observation whose value is far from the rest of the sample. Considering the range of clinically observed values, this data point is not a genuine outlier; instead, it is a consequence of the small number of observations.

Since the proportions of patients preferring transparent material were the same, with a maximum difference of 3.2 % assessed by the binomial test for independent proportions (approximate Miettinen 95% confidence interval = −0.4679 to 0.4679), we can conclude that the preferences were comparable in the study arms. In other words, our findings suggest that patients have preferred transparent material independently of the material used for treatment.

**Figure 18:**
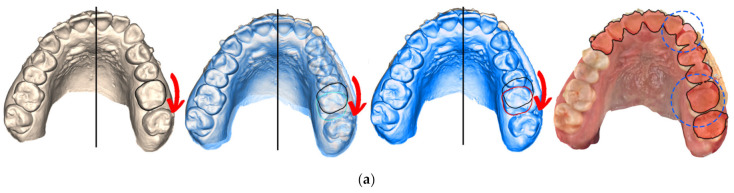

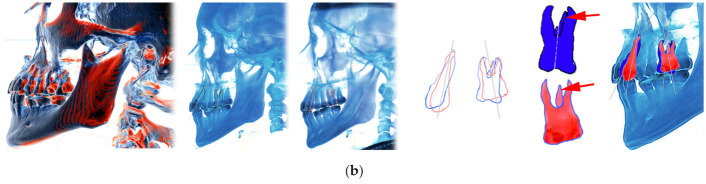
Result of distalization of teeth segments 23–27 with a 3D printed distalizer. (**a**) intraoral scan of one of the results with teeth positions before and after distalization phase. Distorotation of the crown of 26 is shown with a red arrow with an occlusal 2.5–3.0 mm effect circled in blue; (**b**) comparison of original and new (red) CBCT with a visible distal position of teeth in the 2nd quadrant, the second picture is CBCT before treatment, the third picture CBCT after distalization phase followed by contours of teeth 23 and 26 from CBCT, where blue is before and the red contour is a new distalized position.

Clinical evaluation of the 3D printed distalizer effect was obvious and measured digitally on intraoral scan—as shown in Figure 5. In some cases, an option occurred where both intraoral and CBCT scans were available after accomplishment of the distalization with an opportunity to evaluate root positions before and after distalization. Figure 18a shows comparison of the intraoral scan of the upper jaw with distalized teeth 23–27 by means of a 3D printed distalizer bonded on teeth 23 and 26. The achieved result, visible intraorally, could have been analysed on segmented CBCT (Figure 18b) where: the first picture shows a scan before distalization with a red overlay of a new CBCT after distalization superimposed through basis cranii volume matching in the software Invivo Dental 6.5.0 (Anatomage, Santa Clara, CA, USA). This figure shows the resulting orientation of the teeth roots with a more visible 3rd palatal root of the tooth 26 in the final position. This is caused with distorotation movement of this tooth, exposing a palatal root from a lateral view (shown by red arrows).

## 4. Discussion

This paper introduced a personalized distalizer treatment of a Class II malocclusion in adults. Current evidence suggests that orthodontic treatment of such malocclusion should be provided during adolescence. However, early treatment of Class II has been advocated to reduce the risk of incisal trauma—for example, use of a Forsus Fatigue Resistant Device as a non-compliance mandibular advancer after rapid palatal expansion was found to be effective at reducing the sagittal jaw discrepancy, successful at controlling the patient’s unfavourable growth pattern and beneficial for the child aesthetics Goracci, Cacciatore 2017 [68]. This paper proves that non-compliance orthodontic devices are more achievable with personalized 3D printing.

This research has introduced a novel method of personalized orthodontic treatment with a 3D printed distalizer from photopolymer resin. This has been never published before. The closest research on this topic is research published in the journal of clinical orthodontics in 2020 by Simon Graf on the account of CAD/CAM metallic printing of a skeletally anchored upper molar distalizer [69]. This research is about a skeletally borne metallic palatal distalizer. This paper is about a photo-polymeric 3D printed tooth borne vestibular distalizer.

The authors of this paper have recently published research focused on 3D printed orthodontic auxiliary—power-arm [70] using a Finite Element Analysis approach for 3D design. Similar research, also implementing the Finite Element Method to evaluate the tooth movement efficacy of retraction springs made of a new β-titanium alloy, was published with Tamaya et al. [71]. The Finite Element Method with a combination of advanced Artificial Intelligence features like 3D Convolution Neural Networks has a bright perspective for the future of an individualized approach for 3D design of intraoral appliances and is thus effective orthodontic treatment [72]. 

Results showed no significant clinical difference between both tested materials. Despite a white photopolymer being 3D printed on better resolution (50 microns versus 100 microns), there was no clinical impact. Debonding incidents were mostly caused by patient-admitted non-compliance during eating, and material damage was rare. An interesting aspect of this study is that all orthodontic patients’ intraoral situations were monitored remotely with a tele-Health solution—Dental Monitoring, which is judged positively with orthodontists [73] Small differences in material properties had no clinically relevant impact on the distalizers’ performance. Implication of these findings might suggest wider possibility of suitable materials—dedicated dental resins. In 3D modelling of individualized distalizers, we recommend using Open-Source free software like MeshMixer or Blender. In addition, for creation of other orthodontic devices confirmed with the recent publication of Canova et al. 2021, these are preferred with younger clinicians with more extensive digital skills [74].

3D printing in orthodontics now has great focused albeit not limited to orthodontic aligners, and recent research of Zinelis et al. 2021 shows that mechanical properties are dependent not only on the type of resin but also on the method of 3D printing affecting their clinical efficacy [75].

The results can be interpreted from the perspective of previous studies mentioned in the Introduction that photo-polymeric dental resins can be used for 3D printing of an individualized distalizer with the comparable clinical efficiency. From the perspective of extraordinary personalization of intraoral appliances with biocompatible 3D printing, this approach has high potential also in addressing issues linked with cleft therapy with dynamically changing intraoral morphology [76].

Results of research presented by this paper evaluated clinical performance of 3D printed distalizers upon the frequency of their deboning and suggest that there is no significant difference in this parameter between both compared resins. It is known that nanohybrid resin composites, which are well tested, show good clinical/mechanical performance intraorally. Therefore, the bonding failure, as a clinical issue frequently encountered in orthodontic practice, will be rather effect of failure of adhesives than a material breakage [77,78].

The general findings of this paper are that 3D printed biocompatible personalized distalizers can be successfully applied in tooth-borne hybrid treatment in Class II unilateral malocclusions. Results of the comparison of two biocompatible photopolymers showed that small differences in their material properties are not crucial to their clinical performance. Implications resulting from these findings could advocate future wide use of these materials as the dental resins gain better properties and thus intraoral resiliency [79,80].

This research showed preference of patients towards transparent biocompatible material instead of white A2 shade, as the final tertiary objective was to evaluate patient preference of material. Surprisingly, only one patient who was treated with a clear distalizer preferred white appliance “for the next time”. Furthermore, only one of the patients wearing white shade A2 distalizers preferred white distalizer again; the rest would prefer a transparent one if possible. Despite it seeming obvious that transparent material for distalizers was aesthetically more pleasing, the limitation of this consideration is that patients were confronted only with one particular dental shade (A2).

Another limitation of this study is a small set of clinical treatments observed. To widen the set to gather statistically useful numbers of clinical application, it would be necessary to either:

1. reduce strictness of clinical conditions like I. skeletal class patients or bilateral distalization situations;
2. or prolong the research for more years.

As these biocompatible dental resins are continuously updated, prolonged research would bring results that would be already obsolete.

A future contribution of this paper might be relying on the realization of orthodontists that individualization of the orthodontic treatment might be the key for efficient treatment as it was in the past. The incoming smart materials for 3D printing might return the treatment back into their hands. Orthodontics is an emerging field in which polymers have attracted the enormous attention of researchers. In particular, thermoplastic materials have a great future utility in orthodontics, both as aligners and as retainer appliances. The most promising polymeric smart materials are also discussed for their relevance to future orthodontic applications.

With possibilities sourcing from digital workflows and 3D printing, this new method could have been introduced in this paper—empowering orthodontists with a feasible way to create a fully personalized 3D printed distalizer based on a digital intra-oral scan.

To date, no other study has elaborated on the possibilities of biocompatible 3D printing of customized orthodontic auxiliaries like distalizers. No study focused on hybrid Class II treatment with CAT or introduced such a novel method of additive manufacturing of a custom distalizer for every patient. Only recently has a wider range of Class IIa biocompatible dental resins for 3D printing been introduced and fully certified in Europe. An interdisciplinary approach is necessary to invent and establish this prospective approach that includes 3D digital scanning, 3D software modelling, 3D biocompatible printing and orthodontic knowledge of biomechanics.

This paper shall inspire orthodontists to take advantage of intraoral scans, and they do routinely and utilize them in their routines for in-office 3D printing of personalized orthodontic auxiliaries, not necessarily only distalizers. There is another significant aspect of this paper—the encouragement of orthodontists to develop their own customized auxiliaries for hybrid treatments or even in wider clinical context, returning treatment individualization back into their hands with the opportunity to fully personalize the treatment of their patients as they did in the past in the times of legendary “wire-benders”, with every appliance being in-office individualized.

## 5. Conclusions

An in-office 3D printed biocompatible individualized distalizer can be utilized for tooth-borne hybrid orthodontic treatment of Class II unilateral malocclusion.

Upon evaluation of the frequency of debonding of the distalizers on the set of 12 complete orthodontic treatments, we can conclude that there was no significant difference between materials regarding this measure.

Patient evaluation of the most aesthetically pleasing material for the distalizer selected the clear (transparent) material compared to an alternative white (A2) material.

## Figures and Tables

**Table 1:** Material properties of LF Dental clear V2.

Mechanical Properties	Post-Cured Post-Cured Dental LT Clear Resin (V2)	Method
Ultimate Tensile strength	52 MPa	ASTM D638-10 (type IV)
Young’s modulus	2080 MPa	ASTM D638-10 (type IV)
Elongation	12%	ASTM D638-10 (Type IV)
Flexural Strength at 5% Strain	84 MPa	ASTM D790-15 (Method B)
Flexural Modulus	2300 MPa	ASTM D790-15 (Method B)
Hardness Shore D	78 D	ASTM D2240-15 (Type D
Water Absorption	0.54%	ASTM D570-98 (2018)

**Table 2:** Known properties of Denture Teeth Resin (Formlabs, Dentca).

Properties	Post-Cured Denture Teeth Resin (Shade A2)
Density of the material ^1^	1.15 g/cm^3^
Flexural Strength ^2^	90 Mpa
Relative worn volume % of ΔV ^3^	70%

^1^ Method ISO 20795-1; ^2^ Method EN-ISO 20795-1:2013; ^3^ Method ISO/TR 14569-1: 2007.

**Table 3:** Chemical composition of Dental LT clear and Denture teeth Resin (Formlabs, Dentca).

Post-Cured Dental LT Clear Resin (V2)	(% w/w)	Post-Cured Denture Teeth Resin (Shade A2)	(% w/w)
7,7,9-trimethyl-4,13-dioxo-3,14-dioxa-5,12- diazahexadecane-1,16-diyl bismethacrylate	50–75	Proprietary methacrylate monomer	40–60
2-hydroxyethyl methacrylate	10–20	Diurethane dimethacrylate	30–50
Reaction mass of Bis(1,2,2,6,6-pentamethyl-4-piperidyl) sebacate and Methyl 1,2,2,6,6-pentamethyl-4-piperidyl sebacate	<10	Trimethylolpropane trimethacrylate	3–10
Diphenyl (2,4,6- trimethylbenzoyl) phosphine oxide	1–5	Initiator	<3
Acrylic acid, monoester with propane-1,2-diol	0.1–1	Stabilizer	<1
ethylene dimethacrylate	<10	Pigment	<0.7

**Table 4:** Results of clinical evaluation for secondary objectives.

ID	Sex	Age	Distalization Target	Time in Weeks until Target Achieved	Total Failiures ^1^	Material Patient Had	What Material a Patient Would Prefer Next Time
#	[M/F]	[years]	[mm]	[weeks]	#	[CLEAR/WHITE]	[CLEAR/WHITE]
1	F	25	2.50	19	2	CLEAR	CLEAR
2	F	26	3.00	22	2	WHITE	WHITE
3	F	31	3.50	20	0	WHITE	CLEAR
4	M	23	3.00	24	0	CLEAR	WHITE
5	M	29	4.50	33	1	CLEAR	CLEAR
6	F	30	4.00	32	0	CLEAR	CLEAR
7	F	31	2.50	21	0	WHITE	CLEAR
8	F	37	3.00	25	0	CLEAR	CLEAR
9	M	28	3.50	26	0	WHITE	CLEAR
10	F	20	5.00	30	1	WHITE	CLEAR
11	F	21	5.50	36	1	CLEAR	CLEAR
12	F	26	3.00	20	0	WHITE	CLEAR

^1^ Total sum of debonding and breaking.

**Table 5:** Patients’ characteristics and bivariate analysis with Poisson regression.

Variable	Statistics or Category	Group		*p*-Value	Statistical Test
		WHITE	CLEAR		
	*n*	6	6	n.a.	
Age [years]	mean ± SD	27.0 ± 4.10	27.5 ± 5.79	0.866 *	*t*-test
	median (range)	27 (20–31)	27 (21–37)		
Sex	male	1	2	0.591 *	Fisher exact test
	female	5	4		
Side of oral cavity	left	3	3	0.999 *	Fisher exact test
	right	3	3		
Distance to distalization target [mm]	mean ± SD	3.42 ± 0.861	3.75 ± 1.129	0.578	*t*-test
	median (range)	3.25 (2.5–5.0)	3.5 (2.5–5.5)		
Time until target achieved [weeks]	mean ± SD	23.2 ± 4.01	28.2 ± 6.49	0.223	Mann–Whitney test
	median (range)	21.5 (20–30)	28.5 (19–36)		
Total failures ^a^	Mean ^b^ (Poisson CI)	0.5 (0.10–1.46)	0.67 (0.18–1.71)	0.706	Poisson regression
Material preference	white	1	1	0.999	Fisher exact test
	clear	6	6		

Abbreviations: SD—standard deviation, CI—confidence interval. * For descriptive (not inferential) purpose only. ^a^ Total sum of debonding and breaking. ^b^ Expected number of events.

## Data Availability

Data available in a publicly accessible repository that does not issue DOIs. Publicly available datasets were analyzed in this study. This data can be found here [https://docs.google.com/spreadsheets/d/1zfHNou0FCatt7g9R1NFVZhhRL9-JPiza/edit?usp=sharing&ouid=112757014201777395361&rtpof=true&sd=true], accessed on 23 February 2022.

## Abbreviations

3D	Three-Dimensional
AM	Additive Manufacturing
CAT	Clear Aligner Therapy
CDA	Carriere Distalizer Appliance
CMA	Carriere Motion 3D Appliance (Another name for CDA. Table of used abbreviations in alphabetical order.)
STL	Standard Tessellation Language, describes only the surface geometry of a three-dimensional object without any representation of colour or texture

## References

[1] Huang H.-M. (2020). Medical Application of Polymer-Based Composites. Polymers.

[2] Scribante A., Vallittu P.K., Özcan M. (2018). Fiber-Reinforced Composites for Dental Applications. BioMed Res. Int..

[3] Burgard N., Kienitz M., Jourdan C., Rüttermann S. (2021). The Influence of Modified Experimental Dental Resin Composites on the Initial in Situ Biofilm—A Triple-Blinded, Randomized, Controlled Split-Mouth Trial. Polymers.

[4] Grzebieluch W., Kowalewski P., Grygier D., Rutkowska-Gorczyca M., Kozakiewicz M., Jurczyszyn K. (2021). Printable and Machinable Dental Restorative Composites for CAD/CAM Application—Comparison of Mechanical Properties, Fractographic, Texture and Fractal Dimension Analysis. Materials.

[5] Chung Y.-J., Park J.-M., Kim T.-H., Ahn J.-S., Cha H.-S., Lee J.-H. (2018). 3D Printing of Resin Material for Denture Artificial Teeth: Chipping and Indirect Tensile Fracture Resistance. Materials.

[6] Sfondrini M.F., Vallittu P.K., Lassila L.V.J., Viola A., Gandini P., Scribante A. (2020). Glass Fiber Reinforced Composite Orthodontic Retainer: In Vitro Effect of Tooth Brushing on the Surface Wear and Mechanical Properties. Materials.

[7] Chen S., Yang J., Jia Y.-G., Lu B., Ren L. (2018). A Study of 3D-Printable Reinforced Composite Resin: PMMA Modified with Silver Nanoparticles Loaded Cellulose Nanocrystal. Materials.

[8] Tappa K., Jammalamadaka U. (2018). Novel Biomaterials Used in Medical 3D Printing Techniques. J. Funct. Biomater..

[9] Melo M.A. (2020). Bacterial Interactions with Dental and Medical Materials. J. Funct. Biomater..

[10] Mai H.-N., Hyun D.C., Park J.H., Kim D.-Y., Lee S.M., Lee D.-H. (2020). Antibacterial Drug-Release Polydimethylsiloxane Coating for 3D-Printing Dental Polymer: Surface Alterations and Antimicrobial Effects. Pharmaceuticals.

[11] Jindal P., Juneja M., Bajaj D., Siena F.L., Breedon P. (2020). Effects of post-curing conditions on mechanical properties of 3D printed clear dental aligners. Rapid Prototyp. J..

[12] Vasques M.T., Mori M., Laganá D.C. (2020). Three-dimensional printing of occlusal devices for temporomandibular disorders by using a free CAD software program: A technical report. J. Prosthet. Dent..

[13] Milovanović A., Sedmak A., Golubović Z., Mihajlović K.Z., Žurkić A., Trajković I., Milošević M. (2021). The effect of time on mechanical properties of biocompatible photopolymer resins used for fabrication of clear dental aligners. J. Mech. Behav. Biomed. Mater..

[14] Scribante A., Gallo S., Turcato B., Trovati F., Gandini P., Sfondrini M.F. (2020). Fear of the Relapse: Effect of Composite Type on Adhesion Efficacy of Upper and Lower Orthodontic Fixed Retainers: In Vitro Investigation and Randomized Clinical Trial. Polymers.

[15] Sfondrini M.F., Gallo S., Turcato B., Montasser M.A., Albelasy N.F., Vallittu P.K., Gandini P., Scribante A. (2021). Universal Adhesive for Fixed Retainer Bonding: In Vitro Evaluation and Randomized Clinical Trial. Materials.

[16] Proffit W.R., Fields H.W., Moray L.J. (1998). Prevalence of malocclusion and orthodontic treatment need in the United States: Estimates from the NHANES III survey. Int. J. Adult Orthod. Orthognath. Surg..

[17] Ke Y., Zhu Y., Zhu M. (2019). A comparison of treatment effectiveness between clear aligner and fixed appliance therapies. BMC Oral Health.

[18] Rossini G., Parrini S., Castroflorio T., Deregibus A., Debernardi C.L. (2015). Efficacy of clear aligners in controlling orthodontic tooth move-ment: A systematic review. Angle Orthod..

[19] Nanda R., Garino F., Ojima K., Castroflorio T., Parrini S. (2021). Chapter 12: The hybrid approach in Class II malocclusions treatment. Principles and Biomechanics of Aligner Treatment—E-Book.

[20] Bolla E., Muratore F., Carano A., Bowman S.J. (2002). Evaluation of maxillary molar distalization with the distal jet a comparison with other contemporary methods. Angle Orthod..

[21] Grec R.H., Janson G., Branco N.C., Moura-Grec P.G., Patel M.P., Henriques J.F.C. (2013). Intraoral distalizer effects with conventional and skeletal anchorage a meta-analysis. Am. J. Orthod. Dentofacial Orthop..

[22] Ravera S., Castroflorio T., Garino F., Daher S., Cugliari G., Deregibus A. (2016). Maxillary molar distalization with aligners in adult patients a multicenter retrospective study. Prog. Orthod..

[23] Barakat D., Bakdach W.M., Youssef M. (2021). Treatment effects of Carriere Motion Appliance on patients with Class II malocclusion: A systematic review and meta-analysis. Int. Orthod..

[24] Scribante A., Gallo S., Bertino K., Meles S., Gandini P., Sfondrini M.F. (2021). The Effect of Chairside Verbal Instructions Matched with Instagram Social Media on Oral Hygiene of Young Orthodontic Patients: A Randomized Clinical Trial. Appl. Sci..

[25] Rossini G., Parrini S., Deregibus A., Castroflorio T. (2017). Controlling orthodontic tooth movement with clear aligners. An updated systematic review regarding efficacy and efficiency. J. Aligner Orthod..

[26] Garino F., Castroflorio T., Daher S., Ravera S., Rossini G., Cugliari G., Deregibus A. (2016). Effectiveness of composite attachments in controlling upper-molar movement with aligners. J. Clin. Orthod..

[27] Richter D.D., Nanda R.S., Sinha P.K., Smith D.W. (1998). Effect of behavior modification on patient compliance in orthodontics. Angle Orthod..

[28] Lombardo L., Colonna A., Carlucci A., Oliverio T., Siciliani G. (2018). Class II subdivision correction with clear aligners using intermaxilary elastics. Prog. Orthod..

[29] Nedwed V., Miethke R.R. (2005). Motivation, acceptance and problems of invisalign patients. J. Orofac. Orthop..

[30] Rosvall M.D., Fields H.W., Ziuchkovski J., Rosenstiel S.F., Johnston W.M. (2009). Attractiveness, acceptability, and value of orthodontic appliances. Am. J. Orthod. Dentofac. Orthop..

[31] Shah N. (2017). Compliance with removable orthodontic appliances. Evid. Based Dent..

[32] Carano A., Testa M. (1996). The distal jet for upper molar distalization. J. Clin. Orthod..

[33] Antonarakis G.S., Kiliaridis S. (2008). Maxillary molar distalization with noncompliance intramaxillary appliances in Class II malocclusion a systematic review. Angle Orthod..

[34] Carano A., Testa M., Siciliani G. (1996). The lingual distalizer system. Eur. J. Orthod..

[35] Hilgers J.J. (1992). The pendulum appliance for Class II noncompliance therapy. J. Clin. Orthod..

[36] Marure P.S., Patil R.U., Reddy S., Prakash A., Kshetrimayum N., Shukla R. (2016). The effectiveness of pendulum, K-loop, and distal jet distalization techniques in growing children and its effects on anchor unit a comparative study. J. Indian Soc. Pedod. Prev. Dent..

[37] Byloff F.K., Darendeliler M.A., Clar E., Darendeliler A. (1997). Distal molar movement using the pendulum appliance. Part 2 the effects of maxillary molar root uprighting bends. Angle Orthod..

[38] Chaqués-Asensi J., Kalra V. (2001). Effects of the pendulum appliance on the dentofacial complex. J. Clin. Orthod..

[39] Byloff F.K., Darendeliler M.A. (1997). Distal molar movement using the pendulum appliance. Part 1 clinical and radiological evaluation. Angle Orthod..

[40] Ghosh J., Nanda R.S. (1996). Evaluation of an intraoral maxillary molar distalization technique. Am. J. Orthod. Dentofac. Orthop..

[41] Carrière L. (2004). A new Class II distalizer. J. Clin. Orthod..

[42] Martel D. (2012). The Carriere distalizer simple and efficient. Int. J. Orthod..

[43] Rodríguez H.L. (2011). Unilateral application of the Carriere distalizer. J. Clin. Orthod..

[44] Sandifer C.L., English J.D., Colville C.D., Gallerano R.L., Akyalcin S. (2014). Treatment effects of the Carrière distalizer using lingual arch and full fixed appliances. J. World Fed. Orthod..

[45] Nanda R. (1997). Biomechanics in Clinical Orthodontics.

[46] Yin K., Han E., Guo J., Yasumura T., Grauer D., Sameshima G. (2019). Evaluating the treatment effectiveness and efficiency of Carriere Distalizer: A cephalometric and study model comparison of Class II appliances. Prog. Orthod..

[47] Kim-Berman H., McNamara J.A., Lints J.P., McMullen C., Franchi L. (2019). Treatment effects of the Carriere^®^ Motion 3D™ appliance for the correction of Class II malocclusion in adolescents. Angle Orthod..

[48] Khosravi R., Cohanim B., Hujoel P., Daher S., Neal M., Liu W., Huang G. (2017). Management of overbite with the Invisalign appliance. Am. J. Orthod. Dentofac. Orthop..

[49] Mantovani E., Parrini S., Coda E., Cugliari G., Scotti N., Pasqualini D., Deregibus A., Castroflorio T. (2021). Micro computed tomography evaluation of Invisalign aligner thickness homogeneity. Angle Orthod..

[50] Kinzinger G.S., Wehrbein H., Gross U., Diedrich P.R. (2006). Molar distalization with pendulum appliances in the mixed dentition effects on the position of unerupted canines and premolars. Am. J. Orthod. Dentofac. Orthop..

[51] McFarlane B. (2013). Class II Correction Prior to Orthodontics with the Carriere Distalizer. Int. J. Orthod..

[52] Ahmed S.H. (2020). Three dimensional assessment of the long-term treatment stability after maxillary first molar distalization with Carriere distalizer appliance. Life Sci. J..

[53] Wilson B., Konstantoni N., Kim K.B., Foley P., Ueno H. (2021). Three-dimensional cone-beam computed tomography comparison of shorty and standard Class II Carriere Motion appliance. Angle Orthod..

[54] Hamilton C.F., Saltaji H., Preston C.B., Flores-Mir C., Tabbaa S. (2013). Adolescent patients’ experience with the Carriere distalizer appliance. Eur. J. Paediatr. Dent..

[55] Areepong D., Kim K.B., Oliver D.R., Ueno H. (2020). The Class II Carriere Motion appliance: A 3D CBCT evaluation of the effects on the dentition. Angle Orthod..

[56] Gupta D.K., Tuli A., Jain A. (2020). 3D printed material application in orthodontics. Mater. Today Proc..

[57] Eliades T., Zinelis S. (2021). Three-dimensional printing and in-house appliance fabrication: Between innovation and stepping into the unknown. Am. J. Orthod. Dentofac. Orthop..

[58] Guarnieri F.D.F., Briso A.L.F., Ramos F.d.S.e.S., Esteves L.M.B., Omoto É.M., Sundfeld R.H., Fagundes T.C. (2021). Use of auxiliary devices during retreatment of direct resin composite veneers. PLoS ONE.

[59] Technical Data Sheet—Dental LT Clear (V2). https://formlabs-media.formlabs.com/datasheets/2001429-TDS-ENUS-0.pdf.

[60] Formlabs Denture Resins—PKG-RS-F2-DT. https://dental.formlabs.com/store/denture-teeth-resin-1l/.

[61] Form Cure Time and Temperature Settings. https://s3.amazonaws.com/servicecloudassets.formlabs.com/media/Finishing/Post-Curing/115001414464-Form%20Cure%20Time%20and%20Temperature%20Settings/FormCurePost-CureSettings.pdf.

[62] Safety Data Sheet—Dental LT Clear (V2). https://formlabs-media.formlabs.com/datasheets/2001421-SDS-ENEU-0.pdf.

[63] Safety Data Sheet—Denture Teeth. https://formlabs-media.formlabs.com/datasheets/1902185-SDS-ENEU-0.pdf.

[64] Technical Data Sheet—Denture Base and Teeth. https://dental-media.formlabs.com/datasheets/1802131-TDS-ENEU-0.pdf.

[65] Form 2—3D Printer Technical Data Sheet—Online. https://formlabs.com/3d-printers/form-2.

[66] Cha H.S., Park J.M., Kim T.H., Lee J.H. (2020). Wear Resistance of 3D-Printed Denture Tooth Resin Opposing Zirconia and Metal Antagonists. J. Prosthet. Dent..

[67] Espinar-Escalona E., Barrera-Mora J.M., Llamas-Carreras J.M., Solano-Reina E., Rodríguez D., Gil F.J. (2012). Improvement in adhesion of the brackets to the tooth by sandblasting treatment. J. Mater. Sci. Mater. Med..

[68] Goracci C., Cacciatore G. (2017). Early treatment of a severe Class II malocclusion with the Forsus fatigue resistant device. Eur. J. Paediatr. Dent..

[69] Graf S., Vasudavan S., Wilmes B. (2020). CAD/CAM Metallic Printing of a Skeletally Anchored Upper Molar Distalizer. J. Clin. Orthod..

[70] Thurzo A., Kočiš F., Novák B., Czako L., Varga I. (2021). Three-Dimensional Modeling and 3D Printing of Biocompatible Orthodontic Power-Arm Design with Clinical Application. Appl. Sci..

[71] Tamaya N., Kawamura J., Yanagi Y. (2021). Tooth Movement Efficacy of Retraction Spring Made of a New Low Elastic Modulus Material, Gum Metal, Evaluated by the Finite Element Method. Materials.

[72] Thurzo A., Kosnáčová H.S., Kurilová V., Kosmeľ S., Beňuš R., Moravanský N., Kováč P., Kuracinová K.M., Palkovič M., Varga I. (2021). Use of Advanced Artificial Intelligence in Forensic Medicine, Forensic Anthropology and Clinical Anatomy. Healthcare.

[73] Dalessandri D., Sangalli L., Tonni I., Laffranchi L., Bonetti S., Visconti L., Signoroni A., Paganelli C. (2021). Attitude towards Telemonitoring in Orthodontists and Orthodontic Patients. Dent. J..

[74] Federici Canova F., Oliva G., Beretta M., Dalessandri D. (2021). Digital (R)Evolution: Open-Source Softwares for Orthodontics. Appl. Sci..

[75] Zinelis S., Panayi N., Polychronis G., Papageorgiou S.N., Eliades T. (2021). Comparative analysis of mechanical properties of orthodontic aligners produced by different contemporary 3D printers. Orthod. Craniofacial Res..

[76] Urbanova W., Klimova I., Brudnicki A., Polackova P., Kroupova D., Dubovska I., Rachwalski M., Fudalej P.S. (2016). The Slav-Cleft: A Three-Center Study of the Outcome of Treatment of Cleft Lip and Palate. Part 1: Craniofacial Morphology. J. Cranio-Maxillofac. Surg..

[77] Sfondrini M.F., Pascadopoli M., Gallo S., Ricaldone F., Kramp D.D., Valla M., Gandini P., Scribante A. (2022). Effect of Enamel Pretreatment with Pastes Presenting Different Relative Dentin Abrasivity (RDA) Values on Orthodontic Bracket Bonding Efficacy of Microfilled Composite Resin: In Vitro Investigation and Randomized Clinical Trial. Materials.

[78] Miletić I., Baraba A., Basso M., Pulcini M.G., Marković D., Perić T., Ozkaya C.A., Turkun L.S. (2020). Clinical Performance of a Glass-Hybrid System Compared with a Resin Composite in the Posterior Region: Results of a 2-Year Multicenter Study. J. Adhes. Dent..

[79] Alifui-Segbaya F., Varma S., Lieschke G.J., George R. (2017). Biocompatibility of Photopolymers in 3D Printing. 3D Print. Addit. Manuf..

[80] Schuster M., Turecek C., Kaiser B., Stampfl J., Liska R., Varga F. (2007). Evaluation of Biocompatible Photopolymers I: Photoreactivity and Mechanical Properties of Reactive Diluents. J. Macromol. Sci. Part A.

